# Membrane Type 1–Matrix Metalloproteinase/Akt Signaling Axis Modulates TNF-α-Induced Procoagulant Activity and Apoptosis in Endothelial Cells

**DOI:** 10.1371/journal.pone.0105697

**Published:** 2014-08-27

**Authors:** Hiroshi Ohkawara, Toshiyuki Ishibashi, Koichi Sugimoto, Kazuhiko Ikeda, Kazuei Ogawa, Yasuchika Takeishi

**Affiliations:** 1 Department of Cardiology and Hematology, Fukushima Medical University, Fukushima, Japan; 2 Department of Cardiovascular Medicine, Ohara General Hospital Medical Center, Fukushima, Japan; University of Illinois at Chicago, United States of America

## Abstract

Membrane type 1–matrix metalloproteinase (MT1-MMP) functions as a signaling molecule in addition to a proteolytic enzyme. Our hypothesis was that MT1-MMP cooperates with protein kinase B (Akt) in tumor necrosis factor (TNF)-α-induced signaling pathways of vascular responses, including tissue factor (TF) procoagulant activity and endothelial apoptosis, in cultured human aortic endothelial cells (ECs). TNF-α (10 ng/mL) induced a decrease in Akt phosphorylation within 60 minutes in ECs. A chemical inhibitor of MMP, TIMP-2 and selective small interfering RNA (siRNA)-mediated suppression of MT1-MMP reversed TNF-α-triggered transient decrease of Akt phosphorylation within 60 minutes, suggesting that MT1-MMP may be a key regulator of Akt phosphorylation in TNF-α-stimulated ECs. In the downstream events, TNF-α increased TF antigen and activity, and suppressed the expression of thrombomodulin (TM) antigen. Inhibition of Akt markedly enhanced TNF-α-induced expression of TF antigen and activity, and further reduced the expression of TM antigen. Silencing of MT1-MMP by siRNA also reversed the changed expression of TF and TM induced by TNF-α. Moreover, TNF-α induced apoptosis of ECs through Akt- and forkhead box protein O1 (FoxO1)-dependent signaling pathway and nuclear factor-kB (NF-kB) activation. Knockdown of MT1-MMP by siRNA reversed apoptosis of ECs by inhibiting TNF-α-induced Akt-dependent regulation of FoxO1 in TNF-α-stimulated ECs. Immunoprecipitation demonstrated that TNF-α induced the changes in the associations between the cytoplasmic fraction of MT1-MMP and Akt in ECs. In conclusion, we show new evidence that MT1-MMP/Akt signaling axis is a key modifier for TNF-α-induced signaling pathways for modulation of procoagulant activity and apoptosis of ECs.

## Introduction

Matrix metalloproteinase (MMP) proteins, a large family of Zn-dependent endopeptidases, are responsible for degrading a variety of extracellular matrix (ECM) components and for modulating the bioactivity of transmembrane receptors and soluble factors [1], [2]. Degradation of ECM by activated MMPs, such as MMP-2 and MMP-9, plays an integral role in the migration of smooth muscle cells and plaque instability in the pathogenesis of atherosclerosis and consequent hypercoagulability [3], [4]. Membrane type 1–matrix metalloproteinase (MT1-MMP), the first matrix metalloproteinase which was anchored to the cell membrane instead of being soluble, was identified as the fibrinolysin responsible for degrading and remodeling the fibrin matrix during vascular injury and cell recruitment to the vessel wall [3], [5], [6]. It has been reported that MT1-MMP functions as a signaling molecule in addition to a proteolytic enzyme [6], [7]. It has also been reported that molecular links between MT1-MMP and small GTPases, in particular, Rho and Rac explored in cell migration as well as molecular synthesis [8], [9]. Our previous study provides evidence that a lectin-like oxidized low-density lipoprotein receptor-1 (LOX-1)-MT1-MMP axis plays a crucial role in RhoA and Rac1 activation signaling pathways in endothelial dysfunction induced by oxidized low-density lipoprotein (ox-LDL), suggesting that this axis may be a promising target for treating endothelial dysfunction [10]. Furthermore, we reported that MT1-MMP controls thrombin-triggered RhoA and Rac1 activation, resulting in downstream events including Ca^2+^ signaling, reactive oxygen species generation, expressions of tissue factor (TF) and plasminogen activator inhibitor-1 (PAI-1) in human aortic endothelial cells (ECs) [11].

Phosphorylation of protein kinase B (Akt) at two key sites, the activation loop and the hydrophobic motif, activates the kinase and promotes endothelial proliferative dysfunction, leading to apoptosis of ECs, and regulates the balance between cell survival [12], [13]. Akt signaling pathway is also associated with various cellular processes including coagulation and inflammation [13], [14]. Activation of phosphoinositide 3-kinase (PI3K) and its downstream target Akt is essential for TNF-α-induced NF-κB activation as well as decreased TNF-α-induced adhesion molecule expression and monocyte adhesion, which are linked to the development of vascular diseases and induces inflammatory responses in ECs. [15], [16]. Forkhead box protein O1 (FoxO1) is a transcription factor that contributes to physiological processes including Akt-dependent cell proliferation, apoptosis and insulin signaling [17].

In the present study, we hypothesized that MT1-MMP/Akt signaling axis cooperates with NF-κB and FoxO1 phosphorylation in TNF-α-induced signaling pathways of vascular responses, including procoagulant activity and apoptosis of ECs.

## Materials and Methods

### Cell Culture and Reagents

Human aortic ECs were cultured according to the suppliers' instructions (Clonetics Inc., Walkersville, MD, USA and Sanko Junyaku Co., Ltd., Tokyo, Japan). They were used for all experiments after 5 to 10 passages. Recombinant human TNF-α was obtained from R&D systems (Minneapolis, MN, USA), and Akt inhibitor X, a specific pharmacological inhibitor of Akt, was obtained from Merck Millipore (Darmstadt, Germany) [18]. We used a chemical inhibitor of MMP, recombinant TIMP-2 (DAIICHI Fine Chemical Co., Ltd., Toyama, Japan)[10]. We used siRNA to reduce expression of MT1-MMP and Akt, which were purchased from Santa Cruz Biotechnology (Santa Cruz, CA, USA).

### Western blotting

Western blotting was performed as described previously [10], [19]. Cells were lysed with a hypotonic buffer, and the lysate was sonicated. Aliquots containing 20 µg of proteins were subjected to SDS/polyacrylamide gel electrophoresis (10% running gel, 5% stacking gel, PAGEL, ATTO Co., Ltd., Tokyo, Japan) and were then transferred onto polyvinylidene difluoride membranes (EMD Milllipore Co., Ltd., MA, USA). After incubation with blocking solution at room temperature for 30 minutes, the membranes were incubated for 60 minutes at room temperature with primary antibodies in PBS with 5% bovine serum albumin (BSA), and then for 45 minutes with a horseradish peroxidase–conjugated secondary antibody diluted 1∶10000 (Santa Cruz). The signals from immunoreactive bands were visualized by ECL System (GE Healthcare Life Science technologies, FL, USA). Mouse monoclonal antibodies to TF and thrombomodulin (TM), which were obtained from Santa Cruz Biotechnology (Santa Cruz) were diluted 1∶250 in PBS with 5% BSA. Antibody to MT1-MMP (Chemicon International, Inc., Temecula, CA, USA) was diluted 1∶250 in PBS with 5% BSA. Anti-β-actin antibody (1∶1000, Santa Cruz) was used as a loading control.

### Measurement of TF activity

TF activity was analyzed at the luminal surface of adherent endothelial cells and in whole cell lysates of ECs using a colorimetric assay (American Diagnostica GmbH, Stanford, CT, USA), as previously described [20]. TF/FVIIa converted factor X to factor Xa, which was measured by its ability to metabolize a chromogenic substrate. A standard curve with lipidated human TF was performed to assure that measurements were taken in the linear range of detection.

### Measurement of MT1-MMP activity

MT1-MMP activity was performed as described previously [10], [21]. Briefly, cells were lysed with a hypotonic buffer, then sonicated and centrifuged at a high speed (15,000 g) for 30 min. The cell lysates were assessed by a commercially available fluorescent assay kit (SensoLyte 520 MMP-14 assay kit, AnaSpec, San Jose, CA, USA) according to the manufacturer's instructions.

### Small interfering RNA

MT1-MMP and Akt expression was silenced by siRNA as previously described [10]. The MT1-MMP siRNA was obtained from RNAi Co., Ltd. (Tokyo, Japan), and Akt1/2 siRNA from Santa Cruz Biotechnology. HAECs were transfected with double-stranded siRNA in a serum-free medium mixed with oligofectamine (Invitrogen, Carlsbad, CA, USA) according to the manufacturer's instructions. Four hours after transfection, HAECs were incubated in a medium containing 2% fetal bovine serum (FBS) for 48 h.

### Quantitative RT-PCR of MT1-MMP and Akt RNA

Quantitative RT-PCR was performed as previously described [22]. Total RNA was extracted from ECs using the RNeasy Mini Kit (QIAGEN, Hilden, Germany). After treatment with DNase I (Life Technologies, Carlsbad, CA, USA), reverse transcription (RT) was performed using a High Capacity cDNA Reverse Transcription Kit (Life Technologies). Quantitative RT-PCR (qRT-PCR) was performed with TaqMan Gene Expression Master Mix (Life Technologies) and TaqMan Gene Expression Assays for MMP14 (Assay ID: Hs01037009), Akt1 (Assay ID: Hs00178289), HPRT1 (Assay ID: Hs99999909) using Thermal Cycler Dice Real Time System (TP800, Takara, Shiga, Japan). Expression of HPRT1 RNA was used as an internal control.

### Determination of Akt, NF-κB, Caspase-3 and FoxO1 activation

Akt and FoxO1 activation was determined by Western blotting using an anti-phospho-Akt antibody and an anti-phospho-FoxO1 antibody (dilution 1∶250, Biolabs Inc., Ipswich, MA, USA). The anti-phospho-Akt antibody detects endogenous levels of Akt1 only when phosphorylated at Ser473, and also recognizes Akt2 and Akt3. Anti-Akt antibody (1∶250, Cell Signaling Technology) was used as a loading control. We used an anti-phospho-NF-kB p65 antibody (Ser536, dilution 1∶250, Cell Signaling Technology, Inc., Beverly, MA, USA) and an anti-active/cleaved caspase-3 antibody (Asp175, dilution 1∶250, Cell Signaling Technology). Anti-caspase-3 antibody (1∶250, Cell Signaling Technology) was used as a loading control.

### Immunoprecipitation

Membrane and cytosol fractions of ECs were performed as described previously [10], [19]. Briefly, ECs were solubilized in a RIPA buffer (Sigma-Aldrich, St. Louis, MO, USA) and centrifuged at a high speed (15,000 g) for 30 minutes at 4°C to prepare membrane and cytoplasmic fractions. Immunoprecipitation was performed as described previously [10]. Briefly, the fractions were reacted with anti-Akt antibody and anti-phospho-Akt antibody (Biolabs) at a concentration of 1.0 mg/mL. Immunoprecipitated protein was resolved by sodium dodecyl sulfate–polyacrylamide gel electrophoresis, followed by Western blotting. We used the primary antibody against MT1-MMP (Chemicon), which recognized the catalytic domain of MT1-MMP and anti-mouse IgG True blot TM (eBioscience, Inc., San Diego, CA, USA). To show the markers for membrane fractions, cytosol and total lysates for IP: MT1-MMP vs IgG, we used an anti-pan-Cadherin antibody (dilution 1∶250, Santa Cruz) for membrane fractions, an anti-LDHB antibody (dilution 1∶250, Abnova Corp., Taipei, Taiwan) for cytosol and an anti-β-actin antibody (dilution 1∶500, Santa Cruz) for total lysates.

### Fluorescent immunostaining

A mouse monoclonal antibody available to phospho-Akt (Biolabs) was used for immunostaining. We cultured the ECs on chamber slides, and then treated them with or without TNF-α. The ECs were fixed with 4% paraformaldehyde followed by permeabilization with 0.1% Triton in PBS, and then incubated with the antibodies to phospho-Akt and MT1-MMP (Chemicon) diluted 1∶100 in PBS with 5% BSA at room temperature for 60 min. After washing, goat-anti-rabbit Alexa 594 and donkey-anti-mouse Alexa 488 (Life Technologies) were reacted for 60 min. The stained cells in the field were analyzed using a fluorescence microscope and x20 and x10 magnification with scale bars representing 50 µm (BX-53-33NC, Olympus Corp., Tokyo, Japan).

### TUNEL assay

TUNEL assay was performed to analyze DNA fragmentation in ECs undergoing apoptosis as described previously [23]. The cells were fixed with 4% paraformaldehyde for 30 minutes at room temperature, rinsed once with distilled deionized water and incubated with TdT buffer (0.2 M Tris–HCl pH 6.6, 40 mM potassium cacodylate, and 5 mM cobalt chloride). Following that, the TdT buffer containing 0.3 U/ml TdT (Boehringer, Mannheim, Germany) and 10 mM biotinylated 16-dUTP (Boehringer) was added to the cells, which were then incubated at 37°C for 60 minutes. Endogenous peroxidase was inactivated by immersing the cells in 0.3% H_2_O_2_ in methanol for 15 minutes at room temperature. After incubation with 5% BSA in PBS for 1 hour at room temperature to block non-specific binding, the cells were incubated with HRP-labelled goat anti-biotin (1∶100) diluted with 5% BSA in PBS for 2 hours at room temperature, and then washed in PBS. The sites of HRP were visualized by H_2_O_2_ and DAB for 10 minutes. The cells were visualized under a microscope and x100 magnification with scale bars indicating 50 µm (IX73, Olympus Corp.). TUNEL assay images were stored in a computer and the intensity of EC apoptosis staining was quantitatively analyzed by the NIH Image Program (ImageJ).

### Statistical analysis

Statistical analyses were performed using ANOVA with Scheffé's post hoc test if appropriate. A value of *P*<0.05 was considered significant. Data are expressed as means ±SD.

## Results

### Effects of a chemical inhibitor of MT1-MMP, TIMP-2 on TNF-α-induced changes in TF and TM expression in ECs

**Figure 1:**
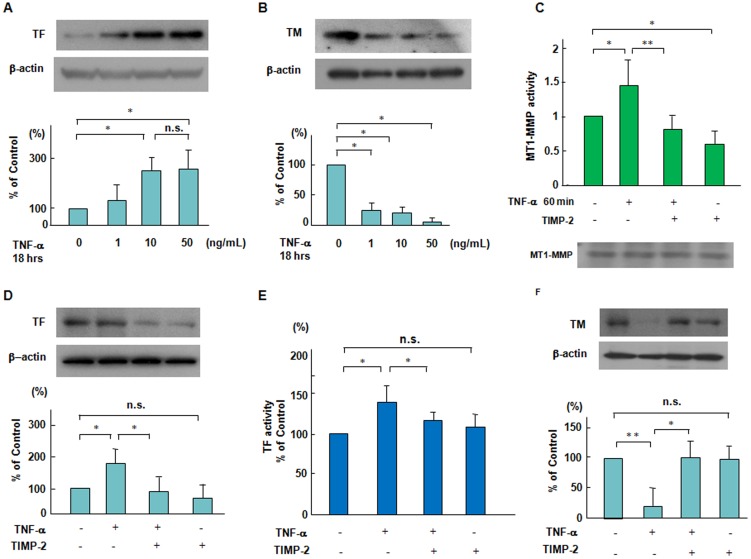
Suppressive effects of TIMP-2 on the changes in TF and TM expression and MT1-MMP activity in response to TNF-α. (A and B) TNF-α-induced TF and TM expression in a dose dependent manner. ECs were treated with TNF-α (1 to 50 ng/mL) for 18 hours. Immunoblots are from an experiment representative of 3 similar experiments. (C) Effect of TNF-α on MT1-MMP activity. ECs were pretreated with 40 nmol/L TIMP-2 for 60 min and then stimulated by TNF-α (10 ng/mL), and thereafter MT1-MMP activity was determined as described in the Methods section. Results are expressed as means±SD of 4 separate experiments.^ *^ *P*<0.05, ^**^ *P*<0.005. The lower panel showed the protein levels of MT1-MMP as determined by Western blotting. (D–F) Effects of TIMP-2 on TNF-α-induced changes in TF and TM expression. ECs were pretreated with TIMP-2 for 60 min and then stimulated by TNF-α, followed by Western blotting and TF activity assay. Results are from an experiment representative of 3 similar experiments. Quantitative results of TF and TM were obtained by densitometry. ^*^ *P*<0.05, ^**^ *P*<0.005.

We first performed Western blot analysis for TF and endothelial anticoagulant TM expression in human aortic ECs in TNF-α stimulation. TM is an endothelial cell membrane glycoprotein that forms a complex with thrombin, converting thrombin from a procoagulant to an anticoagulant enzyme [24], [25]. Eighteen hours after adding TNF-α (1 to 50 ng/mL) to ECs, we determined the expression of TF and TM in ECs. TF antigen levels were increased in a dose-dependent manner up to 10 ng/mL TNF-α, whereas TM antigen levels were decreased in TNF-α stimulated ECs (Figures 1A and B). The activities of secreted MMPs and MT-MMPs are modulated by the tissue inhibitor of metalloproteinases (TIMPs) [26], [27]. TNF-α-increased MT1-MMP activity was blocked by TIMP-2, which is a chemical inhibitor of MT1-MMP (Figures 1C). TNF-α and TIMP-2 had no effect on the protein levels of MT1-MMP within one hour (Figures 1C, lower panel). Furthermore, we showed that 40 nmol/L TIMP-2 inhibited the TNF-α-increased levels of TF antigen and activity as well as the TNF-α-decreased levels of TM antigen in ECs (Figures 1D, 1E and 1F). These findings suggest that MT1-MMP is upstream of TNF-α-induced TF and TM changes in ECs.

### Effects of MT1-MMP siRNA on TNF-α-induced changes in TF and TM expression in ECs

**Figure 2:**
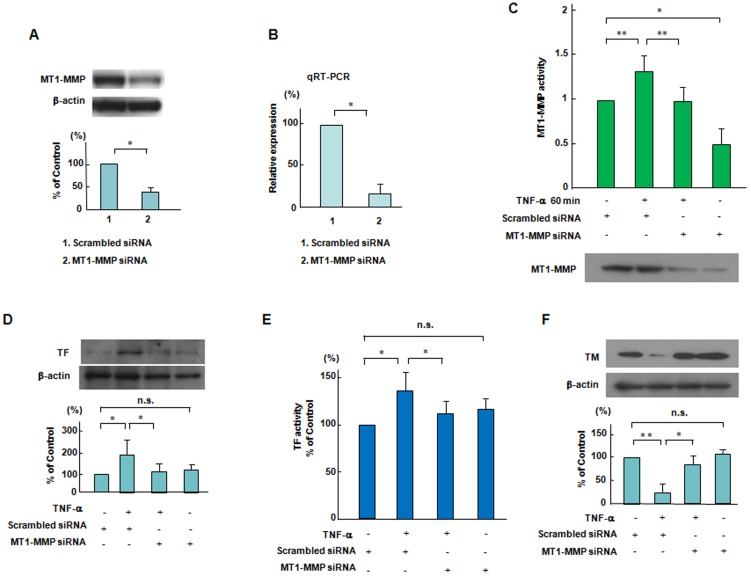
Effects of siRNA-mediated knockdown of MT1-MMP on the changes in TF and TM expression and MT1-MMP activity in response to TNF-α. (A and B) Effects of knockdown of MT1-MMP by siRNA on the MT1-MMP protein and mRNA levels. ECs were transfected with siRNA to MT1-MMP. siRNA-mediated knockdown of MT1-MMP significantly reduced MT1-MMP protein levels and mRNA as compared to the scrambled negative control. Bars are the means±SD of quantitative densitometric analyses from 4 separate experiments. ^*^ *P*<0.05. (C) Effect of knockdown of MT1-MMP by siRNA on MT1-MMP activity caused by TNF-α. ECs were incubated in the presence of TNF-α with or without transfection with siRNA to MT1-MMP, and thereafter the activity of MT1-MMP was determined. Results are expressed as means±SD of 4 separate experiments.^ *^ *P*<0.05, ^**^ *P*<0.005. The lower panel showed the protein levels of MT1-MMP as determined by Western blotting. (D-F) Effects of knockdown of MT1-MMP by siRNA on the TNF-α-induced changes in TF and TM expression. ECs were incubated in the presence of TNF-α with or without transfection with siRNA to MT1-MMP, followed by Western blotting and TF activity assay. Western blot analyses are from an experiment representative of 4 similar experiments. TF activity was measured 6 hour after adding TNF-α (each group, n = 6). ^*^ *P*<0.05,^ **^ *P*<0.005.

Next, we transfected ECs with MT1-MMP siRNA and then added TNF-α to ECs. Figure 2A shows that a siRNA-mediated knockdown of MT1-MMP reduced MT1-MMP protein levels by approximately 65% as compared to the scrambled negative control. MT1-MMP siRNA also significantly reduced MT1-MMP mRNA (Figure 2B). Figure 2C shows that the silencing effects of MT1-MMP siRNA inhibited MT1-MMP activity increased by TNF-α (10 ng/mL) within 60 minutes in ECs. The lower panel of Figure 2C confirmed that MT1-MMP siRNA significantly reduced the protein levels of MT1-MMP in ECs. We investigated that TNF-α-increased levels of TF antigen and activity were inhibited by siRNA-mediated knockdown of MT1-MMP in ECs (Figures 2D and 2E). Figure 2F shows that MT1-MMP siRNA recovered TNF-α-decreased levels of TM antigen in ECs. This indicates that MT1-MMP plays an integral role in mediating a procoagulant state.

### A role of Akt on TNF-α-induced expression of TF and TM in ECs

**Figure 3:**
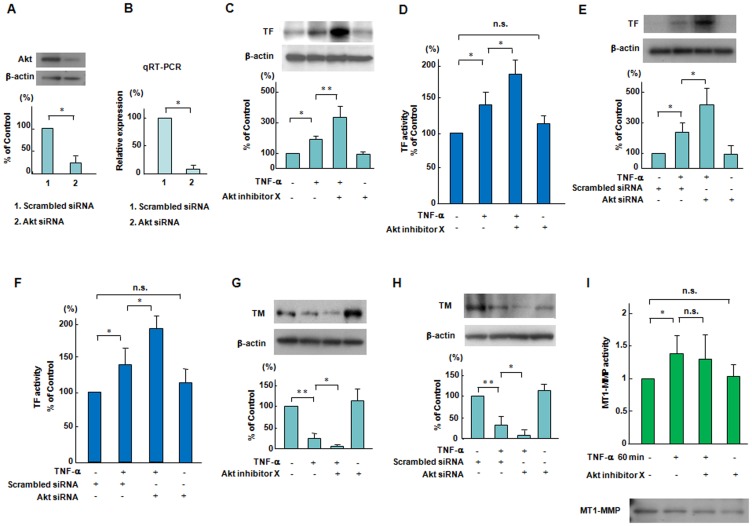
Effects of Akt inhibition on the expression and activation of TF and TM and MT1-MMP activity in response to TNF-α. (A and B) Effects of knockdown of Akt by siRNA on the Akt protein and mRNA levels. ECs were transfected with siRNA to Akt. The siRNA-mediated knockdown of Akt reduced Akt protein and mRNA as compared to the scrambled negative control. Bars are the means±SD of quantitative densitometric analyses from 4 separate experiments. ^*^ *P*<0.05. (C–H) Effects of a specific pharmacological inhibitor of Akt on TNF-α-induced changes in TF and TM expression. ECs were treated with Akt inhibitor X (5 µmol/L) for 60 minutes or Akt siRNA and then stimulated by TNF-α for 18 hours, followed by Western blotting and TF activity assay. Western blot analyses are from an experiment representative of 4 similar experiments. TF activity was measured 6 hour after adding TNF-α (each group, n = 6). ^*^ *P*<0.05, ^**^ *P*<0.005. (I) Effects of a specific pharmacological inhibitor of Akt on on MT1-MMP activity caused by TNF-α. ECs were pretreated with Akt inhibitor X (5 µmol/L) for 60 min and then stimulated by TNF-α, and thereafter MT1-MMP activity was determined as described in the Methods section. Results are expressed as means±SD of 4 separate experiments.^ *^ *P*<0.05. The lower panel showed the protein levels of MT1-MMP as determined by Western blotting.

To selectively inhibit Akt, we used Akt siRNA as well as Akt inhibitor X, a specific pharmacological inhibitor of Akt [18]. An siRNA-mediated knockdown of Akt significantly reduced Akt protein and mRNA as compared to the scrambled negative control (Figures 3A and 3B). Pretreatment with 5 µmol/L Akt inhibitor X and siRNA-medited knockdown of Akt markedly enhanced TNF-α-increased levels of TF antigen and activity in ECs (Figures 3C-3F). Akt inhibitor X and siRNA-mediated the knockdown of Akt reduced TNF-α-increased levels of TM antigen in ECs (Figures 3G and 3H). These results indicate a role of Akt for TNF-α induced signaling pathways downstream of MT1-MMP in the regulation of TF and TM expression in ECs (Figures 3G and 3H). Meanwhile, Akt inhibition did not affect on TNF-α-increased MT1-MMP activity in ECs (Figure 3I). TNF-α and Akt inhibitor X had no effect on the protein levels of MT1-MMP within one hour (Figures 3I, lower panel).

### Effects of TIMP-2 and MT1-MMP siRNA on Akt phosphorylation TNF-α-stimulated ECs

**Figure 4:**
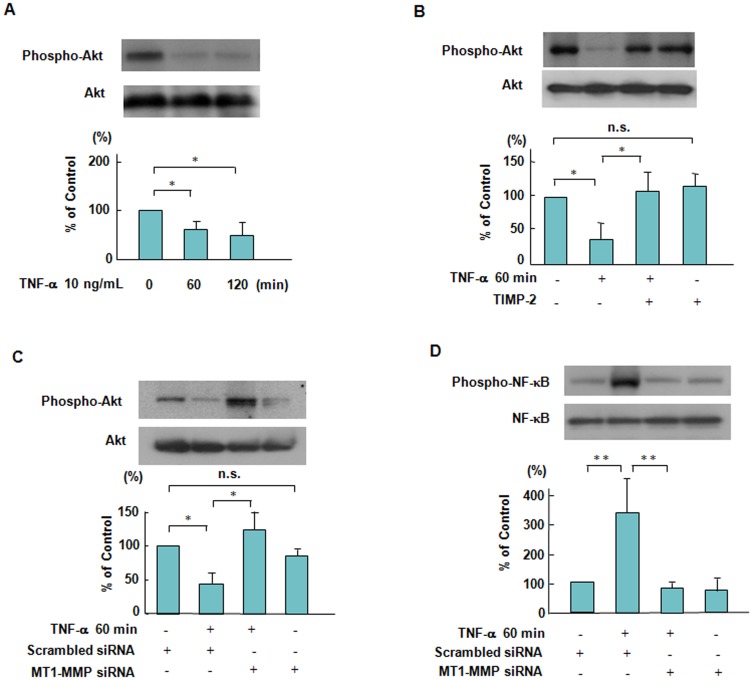
Suppressive effects of TIMP-2 and MT1-MMP siRNA on Akt and NF-κB phosphorylation in response to TNF-α. (**A**) TNF-α-induced Akt phosphorylation in a time dependent manner. ECs were treated with 10 ng/mL TNF-α for 120 minutes, followed by Western blotting. Akt phosphorylation was reduced by approximately 36% within 60 minutes in ECs stimulated by TNF-α. Bars are the means±SD of quantitative densitometric analyses from 4 separate experiments. ^*^ *P*<0.05. (**B**) Effect of TIMP-2 on TNF-α-induced Akt phosphorylation. ECs were pretreated with TIMP-2 and then stimulated by TNF-α. Results are expressed as means±SD of 4 separate experiments.^ *^ *P*<0.05. (**C**) Effect of knockdown of MT1-MMP by siRNA on TNF-α-induced Akt phosphorylation. ECs were incubated in the presence of TNF-α with or without transfection with siRNA to MT1-MMP. MT1-MMP siRNA inhibited the decrease of Akt phosphorylation stimulated by TNF-α in ECs. Bars are the means±SD of quantitative densitometric analyses from 3 separate experiments. ^*^ *P*<0.05. (**D**) Effect of knockdown of MT1-MMP by siRNA on TNF-α-induced NF-κB phosphorylation. ECs were incubated in the presence of TNF-α with or without transfection with siRNA to MT1-MMP, followed by Western blotting. Bars are the means±SD of quantitative densitometric analyses from 4 separate experiments. ^**^ *P*<0.005.

Akt phosphorylation was reduced by approximately 36% within 60 minutes in the ECs treated with 10 ng/mL TNF-α (Figure 4A). TIMP-2 and siRNA-mediated suppression of MT1-MMP inhibited the decrease in Akt phosphorylation in TNF-α stimulated ECs (Figures 4B and C). Our data indicate that MT1-MMP is a critical regulator for Akt-associated signaling in TNF-α stimulation. MT1-MMP has already been reported to trigger NF-κB phosphorylation [28], [29]. To further demonstrate the importance of MT1-MMP, we addressed the possibility that siRNA-mediated suppression of MT1-MMP inhibited NF-κB phosphorylation in TNF-α stimulation, which regulates TF antigen levels and apoptosis of ECs (Figure 4D).

### Molecular interaction of cytoplasmic fractions of MT1-MMP and Akt in ECs

**Figure 5:**
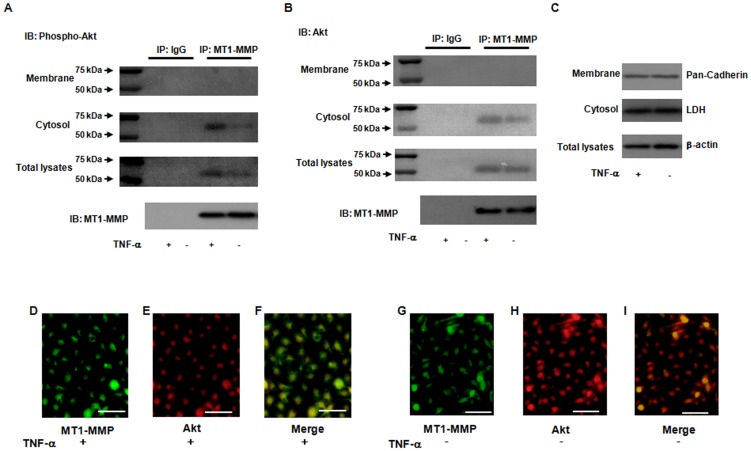
Enhancement of molecular interaction of MT1-MMP and Akt in response to TNF-α. (**A and B**) Formation of a complex of MT1-MMP and phosphorylated Akt as determined by immunoprecipitation. The bands of 60 kDa phospho- and total- Akt were detected in the MT1-MMP-associated immunoprecipitates. Immunoblotting shows that phospho- and total- Akt in membrane and cytosol fractions of ECs was detected in the MT1-MMP-immunoprecipitates in TNF-α stimulation, whereas little or no expression was detected by normal mouse IgG as a negative control antibody. (**C**) Markers for membrane fraction, cytosol and total lysates were determined by Western blotting. Pan-Cadherin and LDH indicate protein markers of membrane fraction, cytosol fraction and total lysates and β-actin, respectively. (**D–I**) Association of MT1-MMP and Akt according to fluorescent immunohistochemistry. ECs treated with or without TNF-α were reacted with the antibodies to phospho-Akt and MT1-MMP, and the stained cells were analyzed by fluorescence microscope. Merged image indicates that MT1-MMP is colocalized with Akt in TNF-α-stimulated ECs. Photomicrographs are from an experiment representative of 4 independent experiments. Scale bars indicate 50 µm (lens x20).

To identify molecular interaction between MT1-MMP and Akt in TNF-α stimulation, we performed immunoprecipitation studies with an initial antibody to MT1-MMP in ECs. As shown in Figures 5A and 5B, the bands of 60 kDa of phosphorylated and total Akt of cytoplasmic fractions of ECs were detected in the MT1-MMP-associated immunoprecipitates in EC treated with TNF-α, but very little or no expression was detectable in the membrane fractions of ECs. Immunoprecipitation study demonstrated MT1-MMP and Akt molecular interactions, mostly in the cytosolic fraction. No MT1-MMP/Akt was found in the membrane fraction. We also performed Western blotting by using the antibodies to pan-Cadherin in the membrane fraction, LDH in the cytosol and β-actin in the cell lysates for IP: MT1-MMP vs IgG (Figure 5C). To further determine molecular interaction between MT1-MMP and Akt, we performed immunohistochemistry for MT1-MMP, Akt and their merged image in ECs treated with or without TNF-α. Immunohistochemistry shows the expression of MT1-MMP and Akt, as well as the merged image (Figures 5D–5I). In particular, Figure 5F shows that TNF-α significantly enhanced the distribution of MT1-MMP and Akt in ECs. These results suggest that molecular interaction of MT1-MMP with Akt in cytosol may contribute to TNF-α-induced signaling pathways in ECs.

### Silencing effects of MT1-MMP siRNA on TNF-α-induced endothelial apoptosis

**Figure 6:**
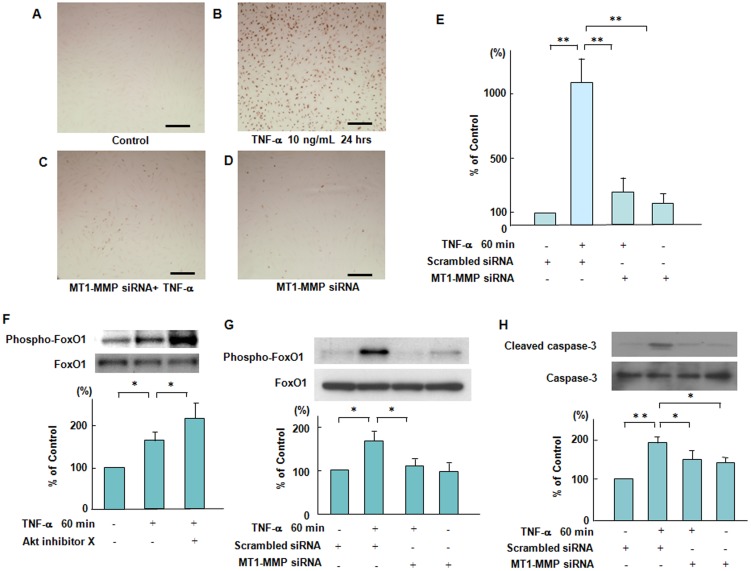
Suppressive effects of MT1-MMP siRNA on TNF-α-induced endothelial apoptosis mediated via the phosphorylation of FoxO1. (**A–D**) Effect of knockdown of MT1-MMP by siRNA on TNF-α-induced endothelial cell apoptosis. ECs were incubated with or without TNF-α for 24 hours. Cell apoptosis was determined by TUNNEL staining. Photomicrographs are from an experiment representative of 4 independent experiments. Scale bars indicate 50 µm (lens x10). (**E**) Quantitative image analysis of cell apoptosis was performed by ImageJ. Data are expressed as means ± SD (n = 3, each group). ^**^ *P*<0.005. (**F**) Effect of a specific pharmacological inhibitor of Akt on TNF-α-induced FoxO1 phosphorylation. ECs were treated with Akt inhibitor X and then stimulated by TNF-α. Bars are the means±SD of quantitative densitometric analyses from 4 separate experiments. ^*^ *P*<0.05. (**G**) Effect of knockdown of MT1-MMP by siRNA on TNF-α-induced FoxO1 phosphorylation. ECs were incubated in the presence of TNF-α with or without transfection with siRNA to MT1-MMP, followed by Western blotting. Immunoblots are from an experiment representative of 4 similar experiments. Bars are the means±SD of quantitative densitometric analyses from 4 separate experiments. ^*^ *P*<0.05. (**H**) Effect of knockdown of MT1-MMP by siRNA on TNF-α-induced caspase-3 activation. ECs were incubated in the presence of TNF-α with or without transfection with siRNA to MT1-MMP, followed by Western blotting. Immunoblots are from an experiment representative of 4 similar experiments. Bars are the means ±SD of quantitative densitometric analyses from 4 separate experiments. *P<0.05, **P<0.005.

**Figure 7:**
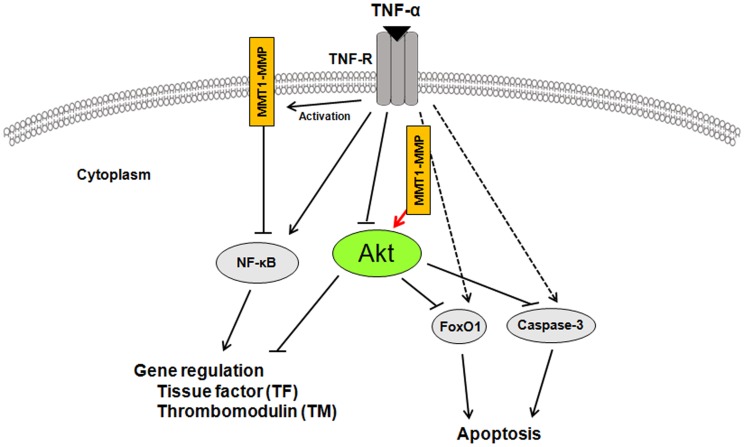
Schematic diagram describing the mechanisms of MT1-MMP/Akt signaling axis in TNF-α-dependent procoagulant activity and apoptosis of ECs. A crucial role of MT1-MMP in Akt-dependednt signaling pathways in TNF-α stimulation. MT1-MMP in the cytoplasm of ECs forms a complex with Akt in the intracellular signaling pathways in TNF-α-stimulated ECs. The interaction between MT1-MMP and Akt regulates TNF-α-induced changes in TF and TM expression in ECs and contributes to endothelial apoptosis through FoxO1 phosphorylation as well as caspase-3 activation.

To investigate the relationship between MT1-MMP and Akt in the mechanism(s) of TNF-α-induced endothelial apoptosis, we performed terminal deoxy (d)-UTP nick end labelling (TUNEL) staining. TUNEL staining showed that 48 hours' treatment with TNF-α induced endothelial cell apoptosis, which was blocked by the silencing of MT1-MMP with siRNA (Figures 6A–6D). Quantitative image analysis of cell apoptosis also confirmed an integral role of MT1-MMP for TNF-α induced endothelial apoptosis (Figure 6E). We next examined whether inhibition of Akt would affect a forehead transcription factor, FoxO1 phosphorylation, which acts as a master switch to control apoptosis [30], [31]. Inhibition of Akt enhanced TNF-α-induced FoxO1 phosphorylation, indicating that Akt is an upstream negative regulator of FoxO1 phosphorylation in TNF-α stimulated ECs (Figure 6F). Moreover, TNF-α-induced FoxO1 phosphorylation was blocked by the silencing of MT1-MMP with siRNA within 60 minutes of TNF-α addition, indicating that MT1-MMP/Akt signaling axis affects TNF-α-induced FoxO1 phosphorylation (Figure 6G). In addition, we showed that TNF-α-induced caspase-3 activation, which controlled endothelial apoptosis, was inhibited by the silencing of MT1-MMP with siRNA (Figure 6H). These results suggest that silencing MT1-MMP with siRNA significantly blocks endothelial cell apoptosis through Akt-dependent regulation of FoxO1 phosphorylation in TNF-α stimulated ECs. Figure 7 described schematically that molecular interaction of MT1-MMP with Akt in the cytoplasm of ECs contributed to TNF-α-induced TF expression through NF-κB phosphorylation and TM expression in ECs and regulated FoxO1 as well as caspase-3 activation in TNF-α-induced apoptosis of ECs.

## Discussion

In the present study, we demonstrated that MT1-MMP/Akt signaling axis modulates FoxO1 and NF-κB phosphorylation in TNF-α-stimulated procoagulant activity and endothelial apoptosis in ECs.

Endothelial cells, which maintain anti-inflammatory and anti-thrombotic surfaces, orchestrate inflammatory and thrombotic responses in the vascular wall [32], [33]. Inflammatory cytokines such as TNF-α are master regulators of vascular proatherogenic changes, which has been linked to endothelial dysfunction in many pathophysiological conditions [32], [34]. TF, a procoagulant protein, can emphasize the intimate link between coagulation and inflammatory processes, whereas TM directly blocks the interaction between thrombin and the procoagulant protein substrates in the pathogenesis of vascular disease [25], [35], [36]. For the first time, we showed that TNF-α induced the increased levels of TF antigen and activity, and reduced the antigen levels of TM in ECs (Figures 1A and 1B).

The membrane-associated MMP, MT1-MMP regulates various cellular functions not only as a pericellular protease but also as a signaling molecule in both physiological and pathological settings [6.7,27]. A chemical inhibitor of MT1-MMP, TIMP-2 inhibited TNF-α-increased MT1-MMP activity in ECs (Figure 1C). TIMP-2 enhanced the increased levels of TF antigen and activity and further reduced the decreased levels of TM antigen in TNF-α stimulated ECs (Figures 1D–1F). Selective siRNA-mediated suppression of MT1-MMP also has similar effects on the expression of TF and TM in TNF-α stimulated ECs (Figures 2D–2F). Our findings suggest that MT1-MMP may be critical for the modulation of procoagulant states in TNF-α stimulation.

The Akt-dependent pathway has been reported to play an important role in vascular hemostasis, including the expression and activity of TF in vascular cells [37]. Our data showed that a specific pharmacological inhibitor of Akt and selective siRNA-mediated suppression of Akt enhanced the TNF-α-induced changes of TF antigen and activity as well as TM antigen in ECs (Figures 3C–3H). In addition, Akt inhibition has no effect on TNF-α-increased MT1-MMP activity in ECs, indicating that MT1-MMP is an upstream regulator for Akt phosphorylation (Figure 3I).

Our previous studies reported that a complex formed with MT1-MMP and LOX-1 plays an integral role in ox-LDL-mediated signaling pathways in ECs, and that MT1-MMP regulates the activation signaling pathways via a receptor for advanced glycation end products in cultured smooth muscle cells [10], [38]. Granulocyte colony-stimulating factor increased MT1-MMP protein and activity in human and murine hematopoietic cells in a PI3K/Akt-dependent manner, suggesting a molecular interaction between MT1-MMP and Akt [39]. In this study, we showed that TIMP-2 and siRNA-mediated suppression of MT1-MMP inhibited the decrease in Akt phosphorylation in TNF-α stimulated ECs. These results indicate that MT1-MMP modulates Akt signaling pathways in TNF-α stimulated ECs (Figures 4A-4C). Akt modulates several cell death-associated factors, including NF-κB and forkhead transcription factors, and prevents cell death [40], [41]. Annabi et al. reported that a MT1-MMP/NF-κB signaling axis regulates COX-2 expression in CD133(+) U87 glioblastoma cells [29]. To further demonstrate the importance of MT1-MMP, we address the possibility that siRNA-mediated suppression of MT1-MMP inhibited TNF-α-induced NF-κB phosphorylation (Figure 4D).

MT1-MMP possesses transmembrane and cytoplasmic domains in addition to extracellular domains, and the cytoplasmic domain of MT1-MMP has an important role in cell invasion and proliferation, indicating that MT1-MMP functions as a signaling molecule [42], [43]. Our immunoprecipitation studies showed that MT1-MMP binds to both total and phosphorylated Akt in the cytosol fractions of ECs treated with TNF-α, but no expression was detectable in the membrane fractions of ECs (Figures 5A and 5B). Our data indicate that MT1-MMP in the cytoplasm of ECs may form a complex with Akt in the intracellular signaling pathway in ECs. This new evidence was strengthened by measuring the protein markers of pan-Cadherin in the membrane fraction, LDH in the cytosol, and β-actin in the cell lysates (Figure 5C). Our immunostaining experiments revealed that MT1-MMP was partially colocalized with Akt in TNF-α stimulated ECs, supporting the idea of MT1-MMP and Akt molecular interactions in TNF-α stimulation (Figure 5F). Further studies are needed to resolve the issue of how interaction between MT1-MMP and Akt induced by TNF-α contributes to MT1-MMP-mediated decrease in Akt phosphorylation. However, our study first describes that molecular interaction between MT1-MMP and Akt in the cytoplasm of ECs contributes to TNF-α-induced signaling pathways in ECs.

We further investigated the integral role of MT1-MMP for the mechanism(s) of TNF-α-induced endothelial apoptosis through Akt-mediated a forehead transcription factor FoxO1 signaling. Our TUNEL staining data show that 48 hours' treatment with TNF-α induced endothelial cell apoptosis, which was blocked by siRNA-mediated suppression of MT1-MMP (Figures 6A-6E). TNF-α induced FoxO1 activation via Akt phosphorylation, which acts as a master switch to control cell apoptosis [41], [44]. Akt inhibition causes FoxO phosphorylation, leading to cell cycle arrest and apoptosis [44]. A specific pharmacological inhibitor of Akt enhanced TNF-α-induced FoxO1 phosphorylation in ECs (Figure 6F). Moreover, siRNA-mediated suppression of MT1-MMP blocked TNF-α-induced FoxO1 phosphorylation as well as caspase-3 activation, indicating that MT1-MMP plays a critical role in the mechanism(s) of endothelial apoptosis (Figures 6G and 6H).

The present study offers new insights into the action of MT1-MMP on Akt-dependent regulation of FoxO1 activation in TNF-α-stimulated ECs. We provide the first evidence that MT1-MMP may play an integral role in TNF-α-induced signaling pathways in cooperation with Akt for the modulation of procoagulant activity and apoptosis of ECs. TNF-α-induced inflammatory responses and procoagulant activity have been implicated in the pathogenesis of vascular diseases including atherosclerosis and myocardial infarction [32]–[36], [45].

Our study suggests that inhibition of the MT1-MMP/Akt signaling pathway may be an attractive strategy for treating endothelial disordered hemostasis in the development of vascular diseases linked to TNF-α-induced inflammation.
